# Vegetarian Diet and Cardiometabolic Risk among Asian Indians in the United States

**DOI:** 10.1155/2018/1675369

**Published:** 2018-02-18

**Authors:** Ranjita Misra, Padmini Balagopal, Sudha Raj, Thakor G. Patel

**Affiliations:** ^1^WVU Public Health Training Center, 3313A, Robert C. Byrd Health Science Center, West Virginia University, Morgantown, WV 26506-9190, USA; ^2^Clinical Nutritionist, Early Intervention, 1901 JFK Blvd, Philadelphia, PA 19103, USA; ^3^Department of Public Health, Food Studies and Nutrition, 562 Falk College, Syracuse, NY 13244, USA; ^4^Department of Medicine, Uniformed Services University of the Health Sciences, Bethesda, MD, USA

## Abstract

Research studies have shown that plant-based diets confer cardiovascular and metabolic health benefits. Asian Indians (AIs) in the US (who have often followed plant-based diets) have elevated risk for chronic diseases such as diabetes, metabolic syndrome, and obesity suggesting ethnic vulnerability that imply genetic and/or lifestyle causative links. This study explored the association between this ethnic group and diabetes, obesity, and metabolic syndrome after controlling for demographics, acculturation, family history of diabetes, and lifestyle and clinical risk factors. The sample comprised of 1038 randomly selected adult AIs in seven US sites. Prevalence and metabolic syndrome was estimated, and obesity was calculated using the WHO Asian criteria. Multivariate analysis included multinomial logistic regression. The mean age and length of residency in the US were 47 and 18.5 years, respectively. The majority of respondents were vegetarians (62%) and educated. A vegetarian lifestyle was associated with females, food label users, respondents with poor/fair current health status, less acculturated, and those who reported their diet had not changed after coming to the US. Vegetarian status was a protective factor and lowered the risk for diabetes but not for metabolic syndrome and obesity in the regression model. Results provide a firm basis for educational programs.

## 1. Asian Indian Population and Vegetarianism

According to the 2013 statistics, the US is home to nearly 3.1 million Asian Indians (AIs), the second largest immigrant Asian after the Chinese Americans [1]. They are among the most socioeconomically successful minority ethnic groups but have higher rates of diabetes and cardiovascular disease when compared to the general US population [2–4], despite the consumption of a traditional predominantly plant-based diet (with the inclusion of some dairy). Research has shown that vegetarians have a lower risk for chronic diseases [5]. However, the role of the Asian Indian vegetarian diet in chronic disease incidence in this population remains unclear. Although a couple of recent studies in the subcontinent have examined associations between types of vegetarian diets and chronic diseases using a large, nationally representative sample [6, 7], there is paucity of data among immigrant Asian Indians.

Contrary to the popular notion of the homogeneity in socioeconomic, cultural, and health characteristics—the “model minority myth” in the sixties and seventies [8, 9]—there is much geographic, linguistic, educational, religious, and socioeconomic heterogeneity in the Asian Indian migrant population today in the US [10, 11]. Several studies based on small, nonrandomized convenience samples have now enumerated the high prevalence rates of noncommunicable diseases (NCDs) among Asian Indians in the US [3, 12–14]. More recently, the Diabetes among Indian American (DIA) study nationwide cohort of Asian Indians brought attention to the high prevalence of diabetes, prediabetes, and metabolic syndrome in this immigrant group and to the importance of early interventions to prevent NCDs [12].

Global health statistics report acculturative dietary and lifestyle changes (e.g., a reduced consumption of traditional foods; a greater reliance on processed and convenience foods high in fat, sugar, and salt; and reduced physical activity) as primary determinants for the high rates of chronic degenerative diseases and associated complications in various ethnic populations, including urban and migrant AIs regardless of geographic location [9, 15]. Rapid socioeconomic developments, a changing food system [16], and migration-related transitions in nutrition, dietary practices, and lifestyles [17] are cited as determinants for these changes. In addition, genetic polymorphisms in AIs [18] and the influence of early maternal/fetal nutrition on body fat [19] are also noted as increasing this chronic disease risk. The well-characterized “South Asian phenotype” [20] equated with metabolic syndrome points to lower adiponectin, higher nonesterified fatty acids and C-reactive protein levels, increased insulin resistance, greater abdominal obesity at a lower body weight, and higher lipoprotein “a” levels that are noted to further accentuate these dramatic shifts in the health status of AIs. The “atherogenic dyslipidemic profile” of this phenotype, well noted in AIs, is implicated in the higher cardiovascular risk, that is, higher concentrations of LDL, hypertriglyceridemia, small dense LDL particles, and lower HDL as well as the preponderance of small, dense dysfunctional proinflammatory HDL-c particles [21]. This unique dyslipidemic profile of AIs coupled with high incidence of insulin resistance is shown to be strongly associated with type 2 diabetes and cardiovascular risk in several studies [22–24].

The vegetarian diet is recognized for conferring numerous health benefits such as reduced morbidity and mortality from chronic degenerative diseases [15]. Contrary to the Western perspective of vegetarianism as an adopted lifestyle by choice in adulthood, vegetarianism in India is a way of life from birth for a third of its population. Derived from religious scriptures and beliefs of Hinduism, Jainism, and Buddhism, the practice of vegetarianism goes beyond the principle of *ahimsa* or nonviolence and is considered a conscious and ethical way of living and eating [25]. Complementary and alternate traditional medical systems such as Ayurveda continue to promote vegetarian practices; a vegetarian diet falls under the domain of biologically based therapies as delineated by the recently named NCCIH (National Center for Complementary and Integrative Health) [26]. Ayurveda describes food as a means to optimize health and promote well-being by holistically addressing mind, body, behavior, and environment [27]. Hence, the traditional Asian Indian vegetarian diet typically uses grains, dairy products, pulses, legumes, and vegetables. Ayurveda has outlined mind and body-altering attributes of foods [27].

Currently, the practice of vegetarianism by Asian Indians often includes widespread prohibition of beef (out of reverence to the cow). Lacto and lacto-ovo vegetarian practices are common; 1.6% of the population follows veganism [6]. Therefore, vegetarian practices vary widely with the extent of religious observances and regional preferences from abstaining from all flesh foods, roots, and tubers to including fish alone [25, 27].

The traditional Asian Indian diet has several health-promoting features. Cereal-legume-pulse combinations are highlights of the cuisine with complementing sources of protein. Native spices such as turmeric, ginger, onion, garlic, chilies, cumin, coriander, and fenugreek provide flavor and aesthetic appeal besides carrying putative medicinal and digestive effects as per Ayurveda [28]. Recent studies endorse some properties as evidenced by the innumerable health benefits of bioactive compounds with potential antioxidant, anti-inflammatory, antimicrobial, antibacterial, and chemopreventive properties [29].

Despite the many touted health-promoting features of the predominantly vegetarian Asian Indian diet, studies report on the increasing rates of NCDs (noncommunicable diseases) such as type 2 diabetes, cardiovascular disease, and metabolic syndrome marked by the raised concentrations of inflammatory markers and cytokines [13, 30].

This study aims to explore the relationship between the consumption of a traditional Asian Indian vegetarian diet and the prevalence of diabetes, obesity, and metabolic syndrome among AIs residing in the United States, after controlling for acculturation, dietary and lifestyle habits, family history of diabetes, knowledge of diabetes and CVD risk factors, and demographic characteristics. Data for this study were derived from the first national population-based investigation of AIs in the US. Diabetes among India American (DIA) study included dietary factors, vegetarian status, demographic, acculturation, and clinical risk factors [12].

## 2. Methodology

### 2.1. Subjects and Data Collection

The sample consisted of 1038 randomly selected Asian Indians, aged 18 years and older, from seven US cities—Houston, TX; Phoenix, AZ; Washington, DC; Boston, MA; San Diego, CA; Edison, NJ; and Parsippany, NJ. The sampling procedure and data collection methodology were reported in prior publications [12, 31]. Participation was voluntary. Survey data were collected via telephone interviews by trained, multilingual AI staff; the response rate was 37%. Although the response rate of 37% is relatively low for an epidemiologic study, it is similar to or higher than the response rate in published health surveys of Asians, Asian Indians, or immigrant ethnic groups [12, 31]. The possible reasons for a 37% response rate might be (a) voluntary participation; (b) reluctance to divulge personal information and provide fasting serum blood; (c) not attributing any value to research studies that require time to complete surveys and fasting blood work; (d) ability to understand the purpose and benefits of the study; and (d) barriers associated with confidentiality, trust, and safety. All participants completed blood work (after a 10-hour fast) and anthropometric measurements (height, weight, and waist circumference). Nonparticipants did not differ in gender, educational level, family history, or smoking status, but were significantly older than participants. Blood samples were centrifuged to separate plasma or serum and shipped on ice to three core laboratories for biochemical analysis (Atherotech Laboratory (Birmingham, AL), Diabetes Diagnostic Laboratory (Columbia, MO), and Translational Metabolism Unit, Baylor College of Medicine (Houston, TX)). This study was approved by the Institutional Review Board of an academic institution.

## 3. Measures

### 3.1. Demographic Variables

Information on age in years, gender, marital status (married, never married, and widowed/separated/divorced), level of education (less than high school, high school, some college, college grad, and post grad/professional), access to conventional health care (health insurance coverage), and self-reported physical health was collected. Income was assessed as a categorical variable with response options from <$10,000 to ≥$150,000. Body mass index (BMI) was calculated from height and weight (BMI = kg/m^2^); nativity status (born in US, yes or no) and length of time in the US were also measured. Current health status was self-reported physical health by respondents and recoded as excellent/very good/good (1) and fair/poor (0).

### 3.2. Acculturation

Acculturation was measured using the nine-question Acculturation Scale for Southeast Asians [32] which assessed two subscales: English proficiency/language and food preferences. Confirmatory factor analysis indicated all items loaded on the subscales and explained 69.3% of the variance. A higher composite score indicated less acculturation (Cronbach's *α* = 0.88).

### 3.3. Dietary and Lifestyle Behavior

Self-reported dietary behavior was calculated from the nutrition subscale (Cronbach's *α* = 0.78) of the revised Health Promotion Lifestyle, Profile II [33]. A higher dietary behavior score was indicative of a healthier dietary behavior. Diet change after immigration to the United States was also obtained (no = 0, yes = 1). Physical activity was calculated from the exercise subscale (9 items that measured the frequency of rigorous or leisure time physical activity; Cronbach's α = 0.83) of the revised Health Promotion Lifestyle, Profile II [33]. The scales ranged from never to routinely with a higher score indicative of physical activity levels. Spirituality status was assessed from the question “do you feel connected with some force greater than yourself?” with response categories “routinely,” “often,” “sometimes,” or “never.” Tobacco usage was dichotomized as nonuser (0) or regular/sometime use of one or more of the following: chewing tobacco or beetle leaves (paan), cigarettes, cigars, and smokeless tobacco (1).

### 3.4. Family History of Diabetes

This was computed from self-reported family history of diabetes. Each occurrence of the chronic condition was rated as 0 = absent and 1 = present and summed to obtain a score that ranged from 0 (none) to 7 (siblings, parents, uncles/aunts, and grandparents).

### 3.5. Fasting Glucose

Fasting capillary glucose (mg/dL) was measured using Accu-Chek Advantage (Roche Diagnostics, Indianapolis, IN). Although fasting serum glucose was collected and stored, analysis indicated abnormal levels with large standard deviations from the capillary glucose for one-third of the respondents. Hence, fasting capillary glucose was used for calculating cardiometabolic syndrome (CMetS) that has been validated in a prior epidemiological study on Indians in India [34].

### 3.6. Clinical Factors

Plasma samples were assayed for triglycerides, Lp(a), homocysteine, hsCRP, LDL, and HDL using the vertical auto profile test at the Atherotech Laboratory (Birmingham, AL) as described previously [35].

## 4. Definitions

### 4.1. Cardiometabolic Syndrome

Cardiometabolic syndrome status was determined according to the International Diabetes Federation (IDF) criteria: presence of central obesity and ≥2 of the following risk factors (RFs): elevated triglycerides (≥150 mg/dL), low HDL (<40 mg/dL in males, <50 mg/dL in females, or specific treatment for these lipid abnormalities), elevated BP (≥130/≥85 mmHg or treatment of previously diagnosed hypertension), and elevated fasting blood glucose (FBG, ≥100 mg/dL or previously diagnosed type 2 DM) [36]. Central obesity was defined using the gender- and ethnicity-specific waist circumference (WC) for Asian Indians [37].

### 4.2. Diabetes Mellitus (DM)

Diabetes was defined as FBG ≥ 126 mg/dL or a self-report of previously diagnosed DM. Impaired fasting glucose was defined as FBG between 100 and 125 mg/dL.

### 4.3. Hypertension

Hypertension was defined as BP ≥ 140/≥90 mmHg or a self-report of previously diagnosed hypertension.

### 4.4. Obesity

Respondents were categorized as normal weight (defined as BMI < 23.0 kg/m^2^), overweight (BMI between 23 and 25 kg/m^2^), and obese (BMI ≥ 25 kg/m^2^) based on the Asian criteria [37]. Waist-to-hip ratio was also calculated.

## 5. Statistics

All analyses were done using the Statistical Program for Social Sciences (SPSS) system (version 23.0). Basic descriptive statistics were obtained for demographic, lifestyle, and clinical factors. Logistic regression analysis was used to examine the association between a vegetarian diet and demographic (age, number of years in the US, gender, education, marital status, income, access to health care, self-rated health, English proficiency, and family history of diabetes) and lifestyle factors (dietary habits, body mass index, spirituality, physical activity, and tobacco use). In addition, logistic regression examined the association between a vegetarian diet and prevalence of diabetes, obesity, and metabolic syndrome, controlling for demographic characteristics, lifestyle factors, and clinical factors (lipoprotein(a), high sensitivity C-reactive protein (hsCRP), homocysteine and triglycerides, HDL, and LDL). The acceptance level for statistical significance was *P* ≤ 0.05.

## 6. Results

### 6.1. Demographic Characteristic and Lifestyle Behavior

The mean age of the subjects was 47.0 ± 12.8 years (Table 1). A majority of respondents were vegetarians (62.7%), male (59%), married (90%), and had access to health care (82%). The modal income was $75,000–$100,000; 15% reported income < $25,000. Mean length of residence in the US was 18.5 ± 11.5 years, and 1.6% were born in the US. Fifty-seven percent reported a family history of diabetes, and 6.6% were current smokers (male predominance).

The mean BMI was 25.4 ± 3.7 kg/m^2^. Using the Asian criteria [37], 25% were overweight (BMI between 23 and 25 kg/m^2^) and 49.8% were obese (BMI ≥ 25 kg/m^2^). However, using the standard criteria by the National Institute of Diabetes and Digestive and Kidney Diseases, 38% were overweight (BMI between 25 and 30 kg/m^2^) and 11% were obese (BMI ≥ 30 kg/m^2^). More than 50% failed to follow the USDA Food Pyramid recommendations for fruit and vegetable consumption (https://www.cnpp.usda.gov/mypyramid), and 65% did not follow the US Surgeon General's recommendation for physical activity (30 minutes a day at least 5 times a week).

The prevalence of chronic diseases was high; rate of diabetes was 17.4%, prediabetes was 33%, metabolic syndrome was 38.2%, and 48% were obese.

## 7. Vegetarian Dietary Habits by Selected Respondent Characteristics

Approximately two-thirds (62%) of AIs reported that they consumed a vegetarian diet, and 50% indicated that their diet has changed after immigrating to the United States. Multivariate logistic regression was performed to examine the association between a vegetarian diet and demographic and lifestyle factors (Table 2). A vegetarian lifestyle was associated with respondents who are less acculturated, not proficient in English, and who reported that their diet had not changed after coming to the US. Asian Indians who self-rated their health status to be fair/poor and were readers of the food label consumed a vegetarian diet. Females were more likely to report a vegetarian diet. Furthermore, respondents with lower education level and who did not consume tobacco in any form reported a vegetarian dietary habit (approaching significance: *P* < 0.10; Table 2).

Demographic variables were significantly associated with a chronic disease risk as expected (not shown in the table). For example, age was positively associated with both risk for diabetes and metabolic syndrome showing chronic disease increased with age; with each year of increase in age, the risk for diabetes increased by 5% (OR = 1.05; 95% CI = 1.02, 1.08) and metabolic syndrome by 3% (OR = 1.03; 95% CI = 1.01, 1.05). Participants with higher incomes also had a higher risk for diabetes (OR = 1.87; 95% CI = 1.06, 3.55) than those with a lower income. Family history of diabetes had a strong association with the risk for diabetes (OR = 3.03; 95% CI = 1.69, 5.42), metabolic syndrome (OR = 1.19; 95% CI = 0.97, 1.45), and obesity (OR = 1.53; 95% CI = 0.94, 2.51). Lack of a healthier dietary behavior (as indicated by a lower intake of fruits and vegetables, grains, protein, and nuts and higher intake of fat) omitting breakfast and not reading food labels showed an increased risk for diabetes while the consumption of fruits and vegetables reduced the risk. High levels of HDL or the good cholesterol reduced the risk for diabetes.

## 8. Are Vegetarians at Lower Risk for Chronic Diseases?

Binary logistic regression was performed to assess the association between a vegetarian diet (primary independent variable) and the prevalence of diabetes, metabolic syndrome, and obesity as the dependent variables, controlling for demographic characteristics, lifestyle factors, and clinical factors (lipoprotein(a), hsCRP, homocysteine, triglycerides, HDL, and LDL) in the model. Multivariate-adjusted odds ratios (OR) are shown in Table 3. As shown in the adjusted model, overall, vegetarian status reduced the risk of diabetes by 44% (OR = 0.55; 95% CI = 0.31, 0.99). However, AIs who consumed a vegetarian diet did not have a reduced risk for metabolic syndrome and obesity (*P* > 0.05).

## 9. Discussion

To the best of our knowledge, this is the first study that explored the metabolic advantages of a vegetarian diet among AIs residing in the United States, after controlling for known confounders such as lifestyle factors, acculturation levels, demographic characteristics, and family history of diabetes. A large percentage (62%) of the sample reported maintaining the traditional practice of consuming a vegetarian diet. A vegetarian diet in this study was described as a lacto-vegetarian diet without eggs, meat, fish, and poultry with a few exceptions that omitted dairy. Our findings confirm that vegetarian status was a protective factor and lowered the risk for diabetes, but not for obesity or metabolic syndrome. Specific dietary macronutrients and micronutrients associated with metabolic benefits in the prevention and treatment of diabetes were not assessed.

Although research has pointed out the higher tendency for AIs in general towards developing type 2 diabetes with a higher risk than the overall US population [17, 21, 23], recent comparisons of urban Indians with their US immigrant counterparts [24] showed a lower prevalence of type 2 diabetes among AI immigrants with a higher prevalence of prediabetes. The results corroborate with these findings. Prevalence of prediabetics in the sample was also high, and while incidence of prediabetes is unknown in this ethnic group, it is very likely that AI immigrants with a prediabetes diagnosis who are physically active and have adopted lifestyle and vegetarian and/or plant-based dietary practices may decrease their dietary risk for progression to diabetes. An increased awareness and knowledge of these health consequences may prompt migrant AIs to make prudent dietary and health-promoting lifestyle food [38] as well as programmed physical activity changes; all of which can contribute to a decreased risk. This is encouraging and needs to be reinforced through public health and nutrition interventions targeted towards Asian Indians.

## 10. Metabolic Syndrome and Obesity

Our study results showed that the vegetarian diet was not protective against metabolic syndrome and obesity. One plausible explanation might point to limitations such as risks like body composition and the nutritional quality of the vegetarian diet consumed that were not assessed in this study. These egregious factors seem to earmark this group towards increased vulnerability to metabolic disease, that is, the unique body composition marked by increasing abdominal obesity and dietary lifestyle practices. The typical AI phenotype has a higher body fat, higher truncal, subcutaneous, and intra-abdominal fat with less lean body mass [20], a feature that is also noted in AI neonates [19]. This coupled with biochemical indicators such as high levels of inflammatory markers, low levels of adiponectin, the coexistence of hyperinsulinemia, insulin resistance, hypertriglyceridemia, abnormal lipid profiles, endothelial dysfunction, and hyperhomocystenemia sets the stage for chronic low-grade inflammation that exacerbates morbidity and mortality. Since muscle mass is an indicator of insulin sensitivity, a lower muscle mass reroutes energy from large carbohydrate meals typical in the Asian Indian diet into hepatic lipogenesis compromising muscle glycogen synthesis. The outcome is atherogenic dyslipidemia [21], a menacing combination that is metabolically linked to insulin resistance, promotes subclinical chronic inflammation, and is strongly associated with type 2 diabetes and cardiovascular disease. Underlying genetic factors such as gene variants and polymorphisms further exacerbate the situation. For example, the ectonucleotide pyrophosphate phosphodiesterase 1 (ENPP1) 121Q variant is implicated in negatively influencing insulin receptor signaling [39] along with the DOK5 gene [40] that increases the risk in both natives in India as well as migrant Asian Indians [39]. Other genetic associations related to chronic diseases noted in this population include the apolipoprotein E gene polymorphisms, the myostatin gene in abdominal obesity and obesity [41], the AMDI variant in homocysteine metabolism that predisposes children to obesity [42], and the PPAR-gamma polymorphisms contributing to nonalcoholic fatty liver disease [43].

Another risk factor is the elevated levels of homocysteine in Asian Indians resulting from a widespread deficiency of vitamin B12 consequent to following a vegetarian diet [44]. High homocysteine levels are implicated as an independent risk factor for cardiovascular disease with deleterious effects primarily affecting the chronic disease process through the oxidative stress [45], modifying protein functionality [46] and subsequent gene expression through altered methylation profiles [47]. Deficiencies and/or altered functioning of the MTHFR (methylenetetrahydrofolate reductase) enzyme is a known mechanism in the elevation of homocysteine levels in the folate vitamin B12 metabolic pathway. Two common gene variants (C677T and A1298C) have been implicated as contributing to defects in the MTHFR gene resulting in metabolic aberrations. A study on the effect of these polymorphisms in more than 400 individuals from different ethnic populations in India based on linguistic lineage and geographic location identified the predominance of the A1298C polymorphism making it a factor that needs further investigation in the quest to identify contributors to the chronic disease paradigm [48]. An examination of the association between homocysteine levels and the C667 T and A1298C polymorphisms in hypertensive patients showed that the presence of either allele C677 T/1298 CC genotypes increased the risk of hypertension. Patients with the MTHFR 1298CC genotype had significantly higher homocysteine levels compared to those with the 1298AA genotype [49, 50].

The dramatic increase of chronic diseases like type 2 diabetes mellitus and cardiovascular disease among Asian Indians seems surprising as there is a long-standing tradition of following a vegetarian diet in this population; a vegetarian plant-based diet has been shown to have lower calories, lower saturated fat content, higher fiber, and similar health-promoting features [51]. Researchers like Dr. Ornish, who have reported success in reversing heart disease, have arguably based the main thrust of success on plant-based vegetarian or vegan diets [52, 53]. This suggests that a comparative study between the Asian Indian vegetarian diet and the vegetarian diet followed by the Seventh Day Adventists in the US could yield vital information on the health effects of the types of vegetarian diets as the latter group has been found to have pronounced benefits [54].

Hence, a few questions arise about the Asian Indian diet such as do they consume an unhealthy vegetarian diet? Or, what is the profile of the current AI vegetarian diet? Singh et al. [15] in a recent review describe certain features of the modern day AI vegetarian diet impacted by a nutrition transition [16]. They highlight the key negative changes as (a) the substitution of refined grains, for example, white rice for whole grains such as brown rice; (b) an emphasis and overconsumption of refined carbohydrates particularly potatoes at the expense of other whole plant foods such as lentils, vegetables, fruits, nuts and seeds, and whole unrefined grains; (c) the increased use of omega-6-rich hydrogenated vegetable oils, palm oil and safflower oil instead of monounsaturated and omega-3-rich olive oil, groundnut oil, rice bran oil, and mustard oil; and (d) the increased consumption of fried/fast/processed foods. This so called “contaminated vegetarianism” [27, 55] profile results from a general lack of awareness of the beneficial qualities of traditional components, a desire to follow Westernized dietary practices as well as the lack of knowledge on modifying traditional diets by altering specific ingredients or replacing cooking methods. On a more positive note, studies on Canadian AI immigrants report a variety of health-promoting behaviors such as an increased consumption of fruits and vegetables as well as increased use of grilling over frying in food preparation [38]. Our study participants who were vegetarian did report a higher vegetable consumption (67.3%). This is a positive behavior that needs to be encouraged. This underscores the importance of making culturally sensitive recommendations to alter negative behaviors while reinforcing/encouraging positive ones to improve health outcomes. Over the past four decades, the practice of vegetarianism among Asian Indians appears to have had fewer followers, both in the older and the younger generations, in the US and among natives in India.

Our results indicated that a vegetarian diet was associated with respondents who were less acculturated and less educated, not proficient in English, and primarily females who also avidly read food labels. Studies on the dietary acculturation of AI immigrants in 1976 and 1986 reported on altered vegetarian status as one of the hallmarks of becoming acculturated [56, 57]. A third of the vegetarians became nonvegetarians within a window period of two months to a year and were eating beef if a person was so inclined by seven years of residing in the US. Females acculturate slower than males; perhaps, as gatekeepers of food and tradition, they hold a higher allegiance to traditional vegetarian dietary practices. Their concern for nonvegetarian ingredients in vegetarian foods may have also prompted greater label reading as was evident in their label use.

The present study did not examine the food components of the diet consumed. Previous dietary acculturation studies reported that altered vegetarian practices in AI immigrants [56, 57] included a change in consumption of ghee, yogurt, Indian bread, rice dishes, and tea from frequent to low moderate upon migration with a concomitant increase in fruit juice, cheese, American breads, dry cereal, soft drinks, and coffee. A study conducted in New York City and Washington in 1999 indicated that vegetarian food patterns and food choices of migrant Asian Indians undergo substantial changes with increasing residence in the US [58]. While consumption of white bread, roots and tubers, vegetable oils, legumes, and tea changed a little, the consumption of fruit juice, chips, fruits, margarine, cola, and alcoholic beverages increased. It is not surprising that when new foods or ingredients are incorporated or substituted for native foods convenience and cost are overarching factors often resulting in calorie dense choices. A lack of nutrition knowledge, inability to judge appropriate portion sizes, being unaware that vegetarian foods can be unhealthy and have large quantities of salt, sugar, and “invisible fats” such as oils, butter, and ghee, increasing the frequency of consumption of “festival/foods eaten on special occasions” that may be calorie dense and fat laden [59], and an easy access to convenient, prepared, frozen native vegetarian entrees in Asian Indian retail outlets are factors fostering these acculturative dietary practices. Participants in a community-based participatory focus group on psychosocial and cultural perceptions had made assumptions that vegetarian diets were low fat or healthy and expressed disbelief that vegetarians could suffer from heart disease [60].

Data on dietary habits of immigrant Asian Indians is limited and is based on small samples in different geographic locations in the US [58, 61–64] but has been useful in providing information on dietary trends in the population. A high-fat intake (32% in migrant AIs), a high omega-6 to omega-3 ratio, the continued use of trans fats as a cooking medium [21], and increased consumption of large carbohydrate meals primarily made up of refined, processed grains with a low fiber content are noted as dietary changes. Our results showed that an unhealthy dietary behavior such as a lower intake of fruits and vegetables, grains, protein, and nuts and higher intake of fat increased the risk for diabetes. While the participants were educated, 43% indicated that they do not read food labels and one-third had a sedentary lifestyle that are likely to have an overriding influence on the metabolic profile and chronic disease progression in AI immigrants. A recent examination of the association between vegetarian dietary patterns and obesity/overweight in a 239 AI member cohort of the Adventist health study showed the potential of whole plant foods, for example, nuts in preventing obesity [15].

Given the alarming increase in NCDs, especially at a younger age, the need for longitudinal population-based epidemiological risk factors for chronic inflammatory conditions such as metabolic syndrome, cardiovascular disease, and diabetes across the life span is underscored. Our results are of great concern for this ethnic population experiencing acculturation and/or assimilation in the US as these diseases are occurring at an early age leading to premature morbidity and mortality [65, 66]. The results also underscore the importance of increasing awareness and educating Asian Indians on the importance of (a) maintaining the authenticity of the traditional Asian Indian vegetarian diet, (b) being mindful of refined processed products, sources and types of fat, and undesirable food preparation and consumption practices, (c) encouraging consumption of whole, unprocessed foods, (d) maintaining physically active lifestyles, and (e) incorporating stress management. Healthcare professionals working with Asian Indian communities should ensure that these messages are targeted across the life span stages so that healthy patterns can be established at an early age. It is equally important to not make assumptions that all vegetarian and/or plant-based diets are healthy diets.

There are currently a few studies among Indians residing in India [6, 67, 68] which highlight macronutrient and micronutrient imbalances in their dietary practices. A large cross-sectional study (*n* = 156, 317 adults in the 20–49 age group) from the National Family Health Survey-3 conducted between 2005 and 2006 showed that lacto-vegetarian, lacto-ovo vegetarian, and semivegetarian diets were associated with lower prospects (~30%) of developing diabetes than pesco-vegetarian and nonvegetarian diets regardless of BMI [6]. Further, men on a lacto-vegetarian and semivegetarian diets had a significantly lower odds ratio of diabetes risk compared to women for whom the consumption of a lactovegetarian diet was protective. Studies on migrant Japanese have confirmed that succeeding generations maintain the intake of foods that are a mark of their cultural identity but often incorporate or substitute accessory high calorie dense foods [69, 70]. For example, second-generation Japanese Americans had higher animal-protein and animal-fat intakes with a diet that resembled more the diet of US than that of Japan [71]. If similar changes and trends are projected among Asian Indians, it could have several ramifications—that the healthful nature of a plant-based diet could become unhealthy with the decreased use of plant foods and the increased reliance on prepared highly refined sugar/cereal and fat foods. The use of stress-released mechanisms such as yoga and meditation has been shown to help in preventing or mitigating cardiovascular diseases [72] prevalent in this ethnic group. The rate of cardiovascular disease in this ethnic group has been estimated to be three times higher than that in Whites [73]. Hence, future studies can examine if Asian Indian vegetarians have the same insulin sensitivity as other ethnic groups who have adopted vegetarian diets. Furthermore, this new immigrant group, despite having some “health-promoting” beliefs such as a predominantly plant-based diet, has shown increased vulnerability towards chronic lifestyle diseases—the Asian Indian Paradox [51, 73]. Hence, additional questions can assess if corrupting dietary factors such as high use of fried foods, refined grains, and sugars and a decreased use of vegetables and whole grains increase their risk despite the “plant-based” features of their cuisine. Today, chronic diseases are taking up a large percentage of health costs, and any information that will help to understand the etiology of chronic diseases can be helpful in their prevention.

A study by Tonstad et al. [74] clearly showed that all variants of vegetarian diets (vegan, lacto-ovo, and pesco-vegetarian and semivegetarian) were associated with substantially lower risk of type 2 diabetes and lower BMI than nonvegetarian diets, and the protection afforded by vegan and lacto-ovo vegetarian diets was the strongest. Why then is this ethnic group (62% of whom were vegetarians) showing increased vulnerability towards metabolic abnormalities?

## 11. Study Limitations

Results of the current study should be considered in context to the following limitations. Vegetarian status, state of diabetes, and lifestyle factors were self-reported and may include social desirability bias that underestimate healthy lifestyle behaviors. Furthermore, the simplistic grouping of a vegetarian and nonvegetarian diets does not capture the variants of the Asian Indian vegetarian diets (vegan, lacto-ovo, and pesco-vegetarian and semivegetarian) to provide a better understanding of the association between a vegetarian diet and risk for NCDs. The present cross-sectional study described a lacto-vegetarian diet without eggs, meat, fish, and poultry with a few exceptions that omitted dairy; the individual components of the diet were not examined. While the study results pointed to the higher prevalence of prediabetes within the population, the actual incidence of diabetes was low. Perhaps, an examination of individual food components under the broad umbrella of a “lacto-vegetarian diet” from a longitudinal perspective may have provided more insight into the role of dietary components in influencing metabolic markers. Future studies should explore the duration and type of vegetarian practice on the incidence of diabetes. Recent explorations of over 800 South Asians in the MASALA study have explored this relationship between the types of vegetarian patterns and metabolic and anthropometric markers [9]. Similar explorations of dietary practices across India [22] point to the role of food choices within a dietary pattern that can make or mar the integrity of the vegetarian practice.

We agree that using capillary blood glucose measurement (rather than serum glucose) is a limitation of this study, and the true prevalence rates might be slightly lower. However, the prevalence of diabetes was high (17.4%) and there was a strong correlation between the average capillary blood glucose levels and HbA1c (*r* = 0.77). Prior studies have also highlighted the reluctance of the Asian Indian participants to give blood for interventional studies. For example, Dr. Chow used to measure blood sugar with a finger stick [34].

Risk for diabetes, obesity, and metabolic syndrome is associated with many factors such as total calorie intake, amount of fat consumption, and amount of meat and vegetable consumption that might modify the risk of NCDs. However, the current study adds to the body of literature as results combine behavioral, anthropometric, and clinical data to present unique perspectives on AIs from a large national study; currently, no national dataset exists for this high-risk (immigrant) ethnic group in the US. While a closer look at macronutrient and micronutrient consumption (e.g., total calorie intake, amount of fat consumption, amount of meat, and vegetables) in AIs can provide a better understanding of excess/deficiencies in AIs, a comprehensive dietary analysis is challenging for Asians in general due to limited ethnic (Asian) food items in currently available nutrition software. Hence, the only way to calculate this will be to enter the ingredient list for each item. Further additional challenges to this process are variations in traditional Indian diet from different geographical regions (e.g., East, West, North, and South). For example, “roti” or Indian bread could be prepared in different ways and calories, fat, and carbohydrate will vary based on the type of roti and its ingredients. Under the circumstances, self-reported dietary behavior of participants, using a reliable and valid nutrition subscale from the revised Health Promotion Lifestyle, Profile II, was considered robust to assess the dietary behavior and diet change for AIs and was used in the current study. Dietary behaviors (consumption of fat, sugar, servings of vegetables, fruits, and carbohydrates and reading food labels) were included in the multivariate regression model, but none attained significance in the model. Evidence from the recent literature suggest that self-reported dietary behavior is associated with a myriad of chronic diseases and outcomes among various racial/ethnic minority groups and even among select groups of non-Hispanic Whites. More specifically, health outcomes associated with dietary behavior have varied widely from hypertension/blood pressure, diabetes, CVD excess weight/obesity, and other health issues [75–88].

## 12. Conclusion

The findings clearly indicated that while a vegetarian diet lowered the risk for diabetes among immigrant AIs in the United States, paradoxically, immigrant AIs have high rates of diabetes, metabolic syndrome, and obesity. A vegetarian lifestyle was associated with females, food label users, respondents with poor/fair current health status, and those who were less acculturated and reported that their diet had not changed after coming to the US. Hence, there exists a gap between knowledge of NCD risk factors and the need for screenings and lifestyle modifications to effectively lower those risks. Plans for future research include enhancing data collection to include the variants of vegetarian diets and the quality of vegetarian diets followed by this ethnic group that negatively impacts body composition and subsequent NCDs to better understand the associations in this high-risk ethnic group using similar population-based studies.

## Figures and Tables

**Table 1:** Demographic characteristics of participants [51].

Variables	Frequency	Percent
Sex		
Male	609	58.7%
Female	429	41.3%
Age	47.0 ± 12.8 years	
Annual household income		
<$25,000	108	14.7%
$25,000–$74,999	236	32.0%
$75,000–$99,999	248	33.6%
≥$100,000+	145	19.7%
Education		
≤High school grad	92	10.5%
College grad or higher	785	89.5%
Have health insurance		
No	150	18.4%
Yes	666	81.6%
Marital status		
Currently married	804	90.2%
Formerly married/never married	87	9.8%
Body mass index	25.2 ± 4.2 kg/m^2^	
English proficiency		
Very well/pretty well	793	88.3%
Not too well/not at all	105	11.7%
Years lived in the US	18.5 ± 11.5 years	
<10 years	252	30.6%
≥10 years	572	69.4%
Tobacco use		
Never	824	92.0%
Sometimes/regular user	72	8.0%
Self-rated health		
Good/excellent	745	84.8%
Fair/poor	134	15.2%
Vegetarian		
Yes	553	62.7%
No	329	31.7%
Diet changed in the US		
Yes	432	51.1%
No	413	48.9%

Tobacco users are respondents who indicated regular or sometimes usage of cigarettes, bidis, chewing tobacco (paan or beetle leaves), smokeless tobacco, and cigars. Spirituality is a subscale of the health promotion lifestyle profile scale (summation of 9 items). Physical activity is a subscale of the health promotion lifestyle profile scale (summation of 9 items). Proficiency in English is assessed by respondents who indicated that they can speak English very well or pretty well. Current health status was assessed by self-reported physical health by respondents. Percentages are valid percentages. Demographic characteristics of the participants have been presented in earlier studies [51].

**Table 2:** Association of vegetarian diet to demographic and lifestyle characteristics.

Variables	Variables	Vegetarian diet		Adjusted odds ratio (95% CI)	*P* value
		Yes	No		
Gender	Male	296 (56.4%)	229 (43.6%)	0.42 (0.25, 0.70)	0.001
	Female	257 (72.0%)	100 (28.0%)		

Age	<50 years	270 (60.8%)	174 (39.2%)	0.94 (0.56, 1.59)	0.822
	≥50 years	278 (65.1%)	149 (34.9%)		

Access to care	No	110 (77.5%)	32 (22.5%)	1.36 (0.69, 2.76)	0.369
	Yes	393 (60.0%)	262 (40.0%)		

Years lived in the US	<10 years	157 (63.1%)	92 (36.9%)	1.02 (0.99, 1.04)	0.211
	≥10 years	350 (62.1%)	214 (37.9%)		

Education	<high school	73 (81.1%)	17 (18.9%)	0.42 (0.15, 1.15)	0.091
	College degree or higher	467 (60.5%)	305 (39.5%)		

Marital status	Not currently married	47 (55.3%)	38 (44.7%)	1.57 (0.72, 3.44)	0.255
	Currently married	501 (63.3%)	290 (36.7%)		

Physical activity^†^	Sedentary	182 (65.7%)	95 (34.3%)	0.80 (0.53, 1.21)	0.31
	Active	355 (60.5%)	232 (39.5%)		

Spiritual^‡^	No	44 (88.0%)	6 (12.0%)	0.95 (0.64, 1.39)	0.78
	Yes	499 (52.6%)	322 (47.4%)		

Feel connected with some force greater than yourself?	Less spiritual	175 (32.2%)	122 (37.2%)	0.68 (0.43, 1.06)	0.090
	Spiritual	368 (67.8%)	206 (62.8%)		

English proficiency^††^	Not very well	86 (87.8%)	12 (12.2%)	2.50 (0.74, 8.41)	0.141
	Well/pretty well	460 (59.4%)	315 (40.0%)		

Self-rated health^¶¶^	Fair/poor	69 (53.5%)	60 (46.5%)	2.01 (1.11, 3.62)	0.021
	Good–excellent	465 (63.7%)	265 (36.3%)		

Income^‡‡^	<$100,000	313 (65.2%)	167 (34.8%)	1.18 (0.76, 1.86)	0.463
	≥$100,000	129 (52.7%)	116 (47.3%)		

Tobacco use^§§^	Never	511 (63.8%)	290 (36.2%)	2.11 (0.91, 4.88)	0.082
	Sometimes/always	32 (47.1%)	36 (52.9%)		

Diet changed in the US	No	294 (62.7%)	112 (37.3%)	0.49 (0.32, 0.77)	0.002
	Yes	224 (61.6%)	202 (38.4%)		

Healthy dietary habits (total)	Never/sometimes	48 (58.5%)	34 (41.5%)	1.69 (0.58, 4.90)	0.338
	Often/always	490 (62.6%)	293 (37.4%)		

Choose diet low in fat & saturated fat	Never/sometimes	301 (60.0%)	201 (40.0%)	0.98 (0.73, 1.31)	0.869
	Often/always	176 (66.7%)	88 (33.3%)		

Limit use of sugar	Never/sometimes	320 (62.6%)	195 (37.4%)	1.04 (0.89, 1.21)	0.627
	Often/always	155 (65.7%)	81 (34.3%)		

Consume 3–5 servings of vegetables per day	Never/sometimes	242 (57.6%)	178 (42.4%)	1.07 (0.78, 1.47)	0.662
	Often/always	272 (67.3%)	132 (32.7%)		

Consume 2–4 servings of fruits per day	Never/sometimes	308 (61.5%)	193 (38.5%)	0.83 (0.62, 1.10)	0.197
	Often/always	185 (64.2%)	103 (35.8%)		

Read food labels	Never/sometimes	241 (57.9%)	175 (42.1%)	1.37 (1.07, 1.75)	0.011
	Often/always	216 (69.0%)	97 (31.0%)		

^†^Physical activity is a subscale of the health promotion lifestyle profile scale (summation of 9 items; range 0 (never) to 3 (always), higher scores indicate a more physically active lifestyle reported by respondents); ^‡^Spirituality is a subscale of the health promotion lifestyle profile scale (summation of 9 items; range 0 (never) to 3 (always), higher scores indicate higher levels of spirituality reported by respondents). Respondents were categorized “physically active” if they responded often or routinely and “physically not active” if responses were never or sometimes. Similarly, respondents were grouped as spiritual if they responded often or routinely for the nine questions and “not spiritual” if responses were never or sometimes; ^††^Proficiency in English is assessed by respondents who indicated that they can speak English very well or pretty well (1) or not very well/not at all (0). ^‡‡^Income was recoded to <$100,000 (0) and ≥$100,000 (1). ^§§^Tobacco users are respondents who indicated regular or sometimes usage of cigarettes, chewing tobacco (paan or beetle leaves), smokeless tobacco, and cigars (1) and nonusers (0). ^¶¶^Current health status was self-reported physical health by respondents and recoded as excellent/very good/good (1) and fair/poor (0). Access to care indicates having health insurance (private, Medicaid, and Medicare) (1) or no health insurance (0). *P* value is based on logistic regression analysis. Demographic characteristics of the participants have been presented in earlier studies [51].

**Table 3:** Association of vegetarian diet to diagnosed diabetes, metabolic syndrome, and obesity in the study population.

Diabetes												
	Diagnosed diabetes		Adjusted for demographics, lifestyle^∗^, and clinical factors^∗∗^		Metabolic syndrome		Adjusted for demographics, lifestyle^∗^, and clinical factors^∗∗^		Obesity		Adjusted for demographics, lifestyle^∗^, and clinical factors^∗∗^	
	No	Yes	Odds ratio (95% CI)	*P*	No	Yes	Odds ratio (95% CI)	*P*	No	Yes	Odds ratio (95% CI)	*P*
*Vegetarian diet*												
No	260	69	0.00 (referent)		201	127	0.00 (referent)		146	242	0.00 (referent)	
Yes	448	105	0.55 (0.31, 0.99)	0.044	337	209	0.89 (0.58, 1.37)	0.613	86	405	0.68 (0.37, 1.23)	0.201

^∗^Age, gender, income, access to care, tobacco use, acculturation, physical activity, diet change after immigration to the US, healthy dietary habits, and family history of diabetes, ^∗∗^Clinical factors included HDL, blood pressure, CRP, homocysteine, and lipoprotein(a).
